# Supplementary figures and images for: Mre11 exonuclease activity promotes irreversible mitotic progression under replication stress
Source: Life Sci Alliance. 2022 Mar 15;5(6):e202101249. doi: 10.26508/lsa.202101249 (PMC8924007; doi:10.26508/lsa.202101249)

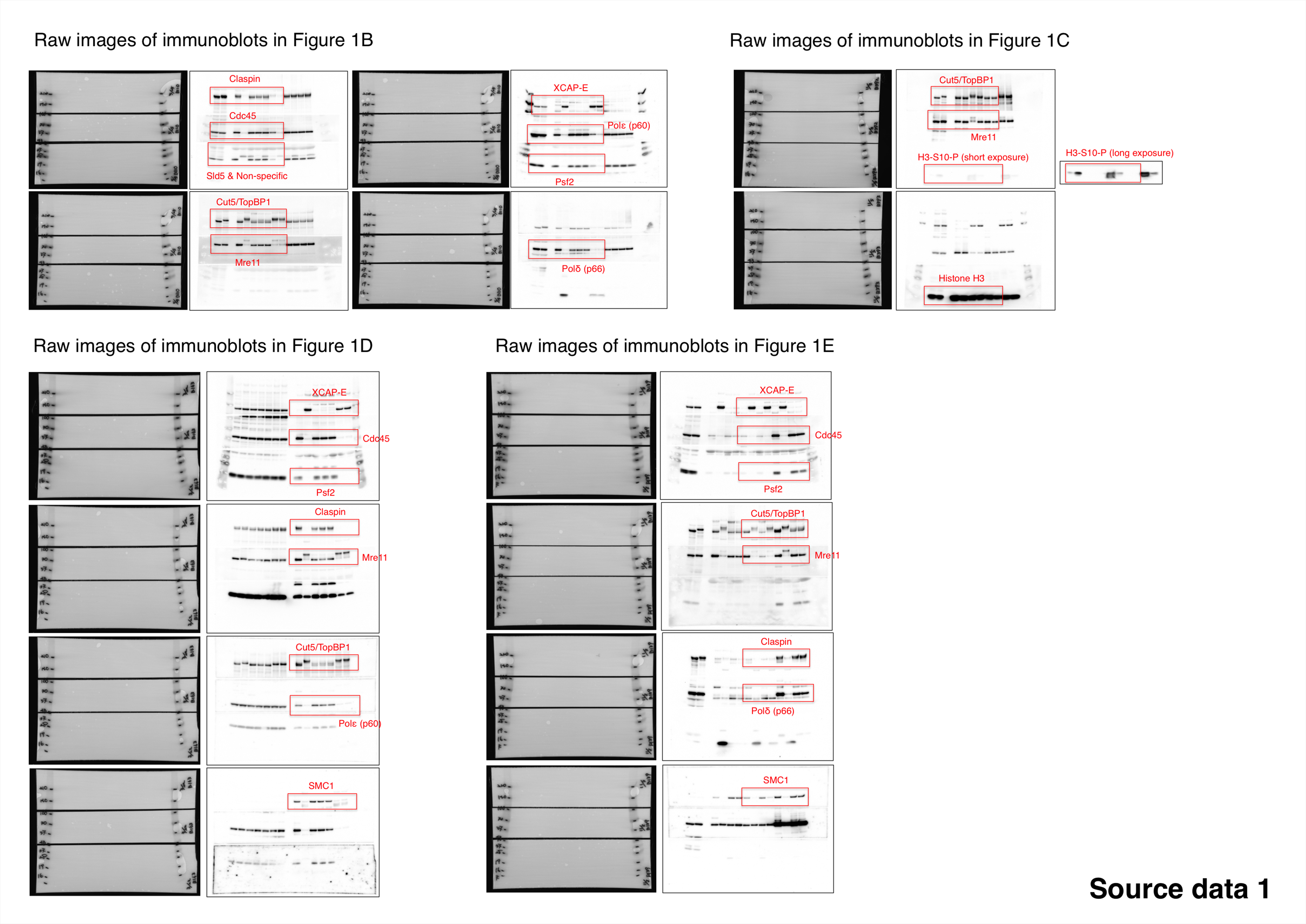

Supplement: Supplementary file 1 [file LSA-2021-01249_SdataF1.tif]

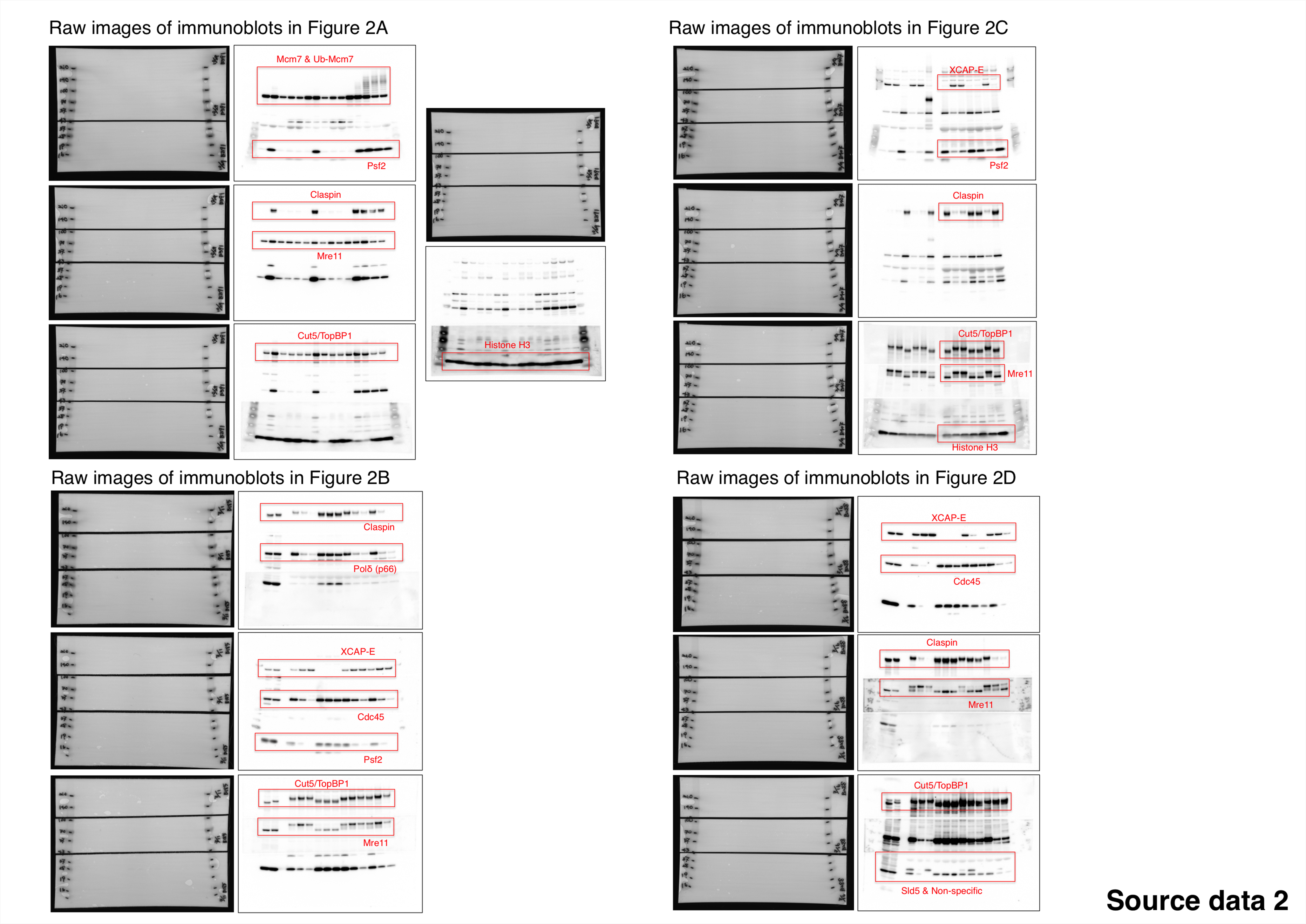

Supplement: Supplementary file 2 [file LSA-2021-01249_SdataF2.tif]

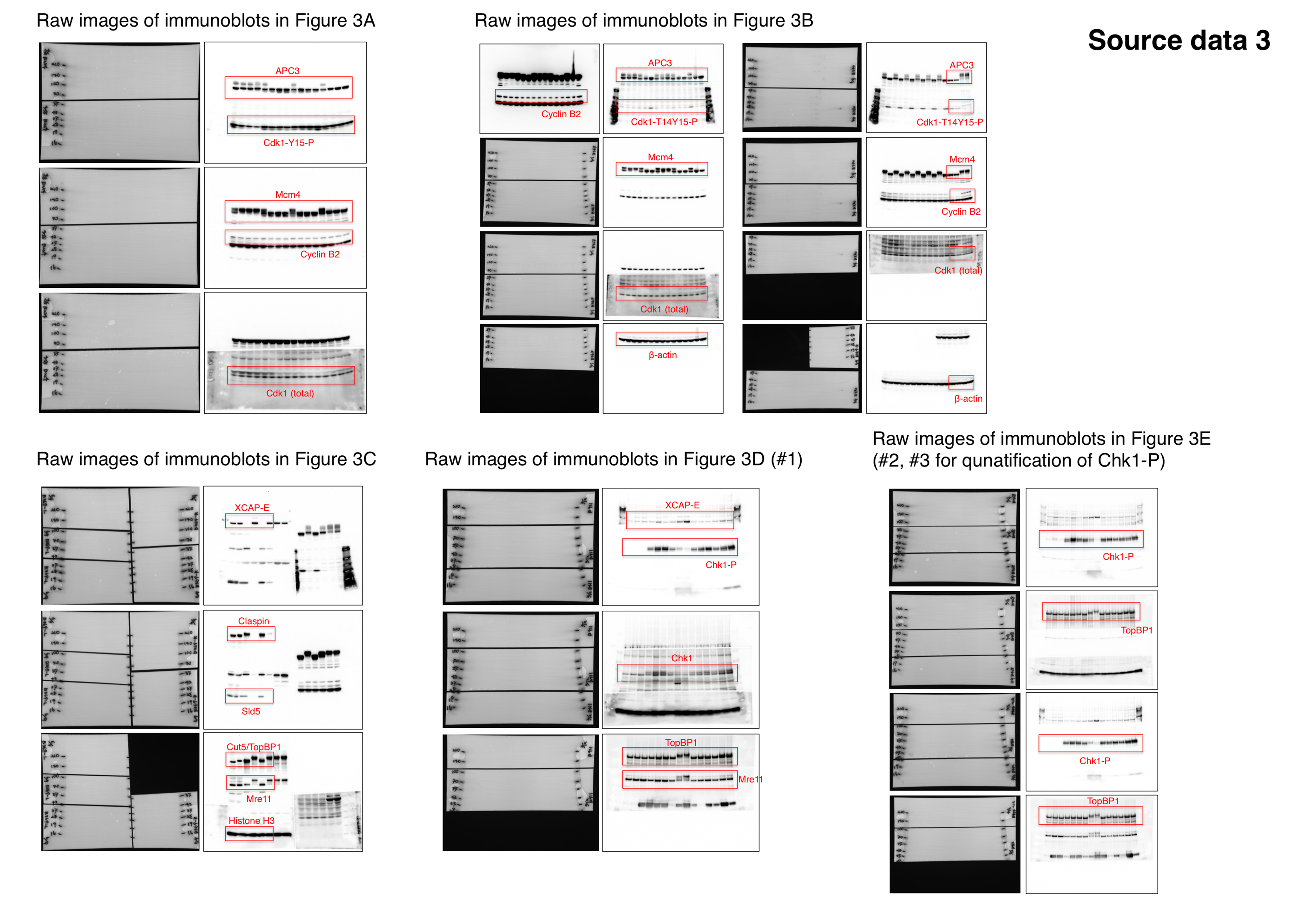

Supplement: Supplementary file 3 [file LSA-2021-01249_SdataF3.tif]

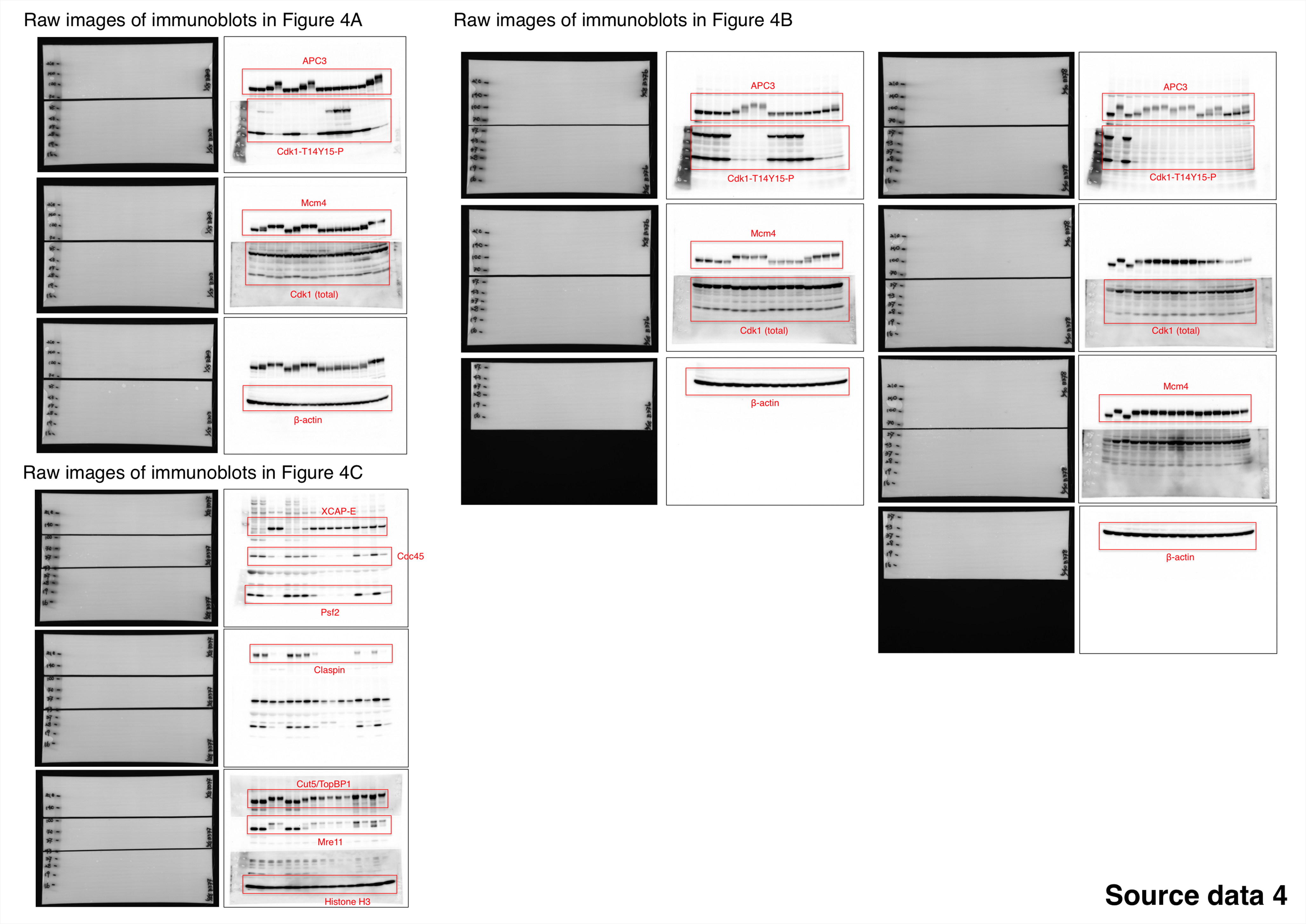

Supplement: Supplementary file 4 [file LSA-2021-01249_SdataF4.tif]
